# Research on Subsurface Electrical Structure Based on a Dense Geomagnetic Array in Southern Yunnan

**DOI:** 10.3390/s24196221

**Published:** 2024-09-26

**Authors:** Xiaoyu Shen, Yujia Cao

**Affiliations:** 1 School of Integrated Circuits and Communications, Suzhou Vocational Institute of Industrial Technology, 1 Zhineng Avenue, Suzhou International Education Park, Wuzhong Avenue, Suzhou 215104, China; 2 Institute of Geophysics, China Earthquake Administration, No. 5 Minzudaxue Nanlu, Haidian District, Beijing 100081, China; caoyj@cea-igp.ac.cn

**Keywords:** dense geomagnetic observation array, southern segment of the Xiaojiang Fault Zone, subsurface resistivity, electrical structure

## Abstract

The electrical resistivity of subsurface rocks is one of the important sensitive parameters characterizing the internal physics of the Earth. Currently, research on subsurface electrical structures using geomagnetic sounding methods primarily focuses on two approaches: the first is based on observations from a few geomagnetic stations, which have low spatial resolution and cannot effectively describe the distribution of anomalies; the second is based on mobile geomagnetic observations, which have low temporal resolution and cannot promptly reflect anomalies. To address these issues, this study deployed a dense geomagnetic array for long-term observation in the southern segment of the Xiaojiang Fault Zone in the Yuxi area of southern Yunnan. This setup aims to promptly capture seismic magnetic anomalies, providing more data support and fundamental information for short-term earthquake prediction. Based on the long-term observation data from the dense array, the study of the subsurface electrical structure is carried out. The results indicate that during the observation period, which was seismically quiet, the regional subsurface electrical structure remained stable. A large-scale subsurface low-resistivity body was observed in the region, and the electrical structures at the two ends of the southern segment of the Xiaojiang Fault Zone were found to be completely different.

## 1. Introduction

In the field of Earth sciences, the geomagnetic field is one of the few geophysical fields that can reflect the properties of deep Earth media, as well as the migration of materials and energy. In terms of earthquake monitoring, as research into the relationship between earthquakes and geomagnetic phenomena deepens, scholars have found that the geomagnetic field may exhibit corresponding anomalous changes during the process of earthquake preparation and occurrence [1,2].

Geomagnetic sounding is based on electromagnetic induction theory, using the natural electromagnetic field as the source. It investigates the deep subsurface electrical structure by analyzing observed data from the surface magnetic field. The geomagnetic field includes a component of variable magnetic fields, which are mainly composed of an external source field generated by the space current system, and an internal source field generated by induction within the Earth. In mid-latitude regions, the external source field causing short-period variations in the geomagnetic field is approximately uniform over a wide area [3]. The intensity and distribution of the induced magnetic field, however, are influenced not only by the external source field but also by the electrical structure of the Earth’s internal media. A wealth of observation facts show that even among stations located very close to each other, there are significant differences in short-period geomagnetic variations [4,5,6,7,8,9]. These differences are referred to as short-period geomagnetic variation anomalies. The anomalies actually reflect the lateral heterogeneity of the subsurface conductivity structure and are also known as resistivity anomalies [10].

Resistivity anomalies are influenced not only by factors such as rock composition and temperature but also by other geophysical phenomena, including seismic wave low-velocity zones and geothermal flux anomalies. Therefore, studying resistivity anomaly zones is of great significance for exploring the transformation of the composition and structure of the lithosphere, geodynamic processes, source mechanisms of earthquakes, and the relationship between earthquakes and geomagnetic phenomena.

However, existing geomagnetic station networks are typically sparse and consist of only a few stations. Consequently, the analysis of geomagnetic anomalies related to earthquakes often relies on data from only one station near the epicenter. The reliability of the anomalies is often questionable when the interfering data cannot be eliminated. Furthermore, due to the limited range of precursory geomagnetic anomaly signals extracted using methods such as polarization, a 90% probability of detecting precursory ULF wave anomalies exists only when the relationship between epicentral distance R and the magnitude M satisfies 0.025R ≤ M−4 [11,12]. Therefore, only a dense measurement point array can effectively meet the requirement of having multiple observation points within the epicentral distance range.

Currently, geomagnetic observations are typically conducted by establishing geomagnetic stations, including geomagnetic reference stations and basic geomagnetic stations. While these stations can provide long-term continuous geomagnetic observation data, the distance between stations is too large. The distance between basic stations is generally about 200 km, and between reference stations it is about 600 km, resulting in low spatial resolution and insufficient capability to effectively describe the distribution and changes of localized geomagnetic anomalies in earthquake-prone areas. Meanwhile, dense geomagnetic observations in key earthquake-monitoring areas are currently mainly conducted through mobile geomagnetic measurements, typically performed up to four times a year. Moreover, these measurements often only observe the total magnetic field strength and do not capture information on variations in the magnetic field, affecting the timeliness of obtaining geomagnetic anomaly information related to earthquakes. To address the limitations of existing observation methods, geomagnetic observations are increasingly utilizing buried instruments and implementing dense deployments within specific regions.

Obtaining geomagnetic field variation information with sufficient spatial and temporal resolution is essential for earthquake monitoring studies. Therefore, it is crucial to establish a dense geomagnetic observation array in targeted areas to monitor geomagnetic field changes in seismically hazardous zones such as active fault zones. This approach will help capture geomagnetic anomaly information in time, provide more data support and foundational information for short-term earthquake forecasting, and enable effective tracking and monitoring of seismic activities in earthquake-prone and key monitoring areas.

Based on the world’s largest dense geomagnetic observation array in the southern segment of the Xiaojiang Fault Zone in Yuxi, southern Yunnan, China, this paper utilizes the geomagnetic sounding method to conduct a preliminary applied study of the subsurface electrical structure in the observed area.

## 2. Observation Area and Data Collection

The collision between the Indian and Eurasian plates has caused the rapid uplift of the Tibetan Plateau and intense orogenic movements in the surrounding areas [13]. Along the eastern boundary of the southern half of the Sichuan–Yunnan rhombic block, large faults such as the southern segment of the Xiaojiang Fault Zone, the Qujiang Fault, the Jianshui Fault, and the Honghe Fault are distributed, which are earthquake-prone and seismically hazardous zones that require intensified monitoring.

The Xiaojiang Fault Zone is a significant active fault zone in Yunnan. It is located in the southern segment of the Xianshuihe–Xiaojiang Fault Zone, which forms the northeastern to eastern boundary of the Sichuan–Yunnan rhombic block, with an overall basic SN strike and a modern activity mode dominated by left-trending strike-slip [14,15]. The Xiaojiang Fault Zone and its surrounding area are marked by low earthquake frequency but high-intensity events that cause significant damage. Since 1500, there have been 15 earthquakes with a magnitude of 6.0 or higher near the Xiaojiang Fault Zone, including six with magnitudes between 6.0 and 6.9, eight with magnitudes between 7.0 and 7.9, and the strongest being the 1833 Songming Ms 8.0 earthquake.

In addition to the Xiaojiang Fault Zone, the Qujiang Fault, Jianshui Fault, and Honghe Fault are three significant northwest-trending faults in the region. These faults are approximately equidistantly distributed from north to south. The southern segment of the Xiaojiang Fault Zone intersects and interacts with these northwest-trending fault zones, creating a complex wedge-shaped fault block structure in the southern segment of the Xiaojiang Fault Zone and its surrounding areas [16,17].

The southern segment of the Xiaojiang Fault Zone and its surrounding areas are part of the key earthquake monitoring and defense zone in southern Yunnan, China. Therefore, this study selects the region between 23.4° N to 25° N latitude and 102° E to 103.5° E longitude as the observation area for the dense geomagnetic array.

**Figure 1:**
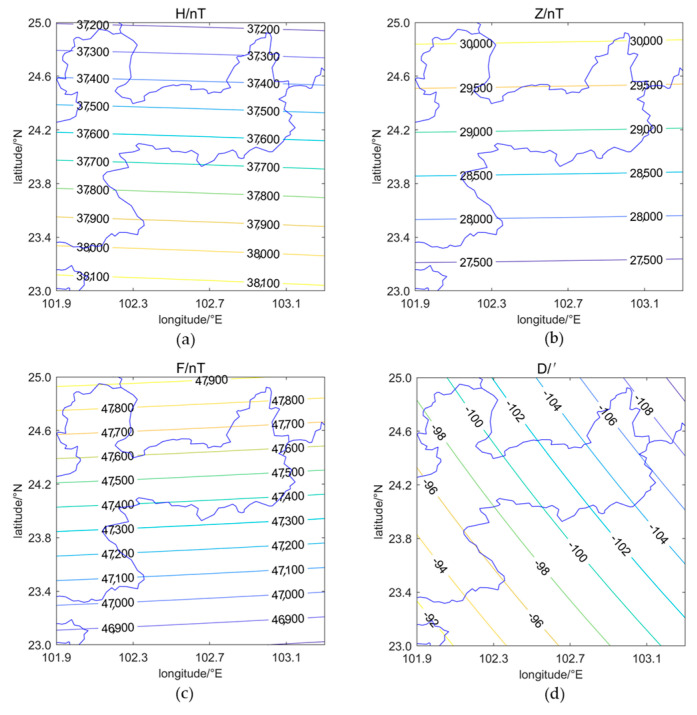
(**a**) Magnetic field horizontal intensity. (**b**) Magnetic field vertical intensity. (**c**) Magnetic field total intensity. (**d**) Magnetic field declination.

According to the latest International Geomagnetic Reference Field Model IGRF-13 [18] proposed by the International Association of Geomagnetism and Aeronomy (IAGA) in 2019, the contour maps of the magnetic field components in the observation region are shown in Figure 1. It can be seen that, in the observation area, the contour lines of the horizontal intensity, vertical intensity, and total intensity of the magnetic field are primarily aligned along latitudes, while the contour lines of the magnetic declination are roughly aligned in a north–south direction.

**Figure 2:**
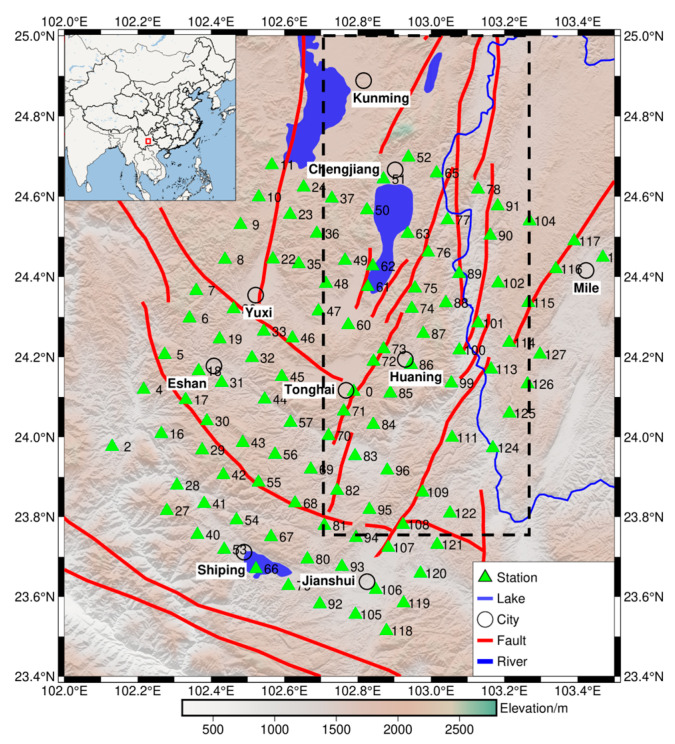
Layout of array measurement points (The red box shows the regional location of the observation area in China).

From Figure 1, it can be seen that the geomagnetic field in the observation area is clearly distributed along the latitude and longitude. Therefore, the dense array can be designed to be quasi-uniformly distributed according to geographical latitude and longitude. Considering the spatial resolution requirement of approximately 10 km between measurement points in the array, the distribution and orientation of faults in the observation area, and the local environment at the measurement points, the dense geomagnetic array measurement points are shown in Figure 2. In Figure 2, the black dashed line box outlines the area of the southern segment of the Xiaojiang Fault Zone, the red solid lines represent the faults, and the green triangles indicate the measurement points. The 110 measurement points are quasi-uniformly distributed along the latitude and longitude, covering an observation area of approximately 130 km by 110 km. This area includes the southern segment of the Xiaojiang Fault Zone, the Qujiang Fault, and the Jianshui Fault; this is beneficial for monitoring seismic electromagnetic anomaly signals.

**Figure 3:**
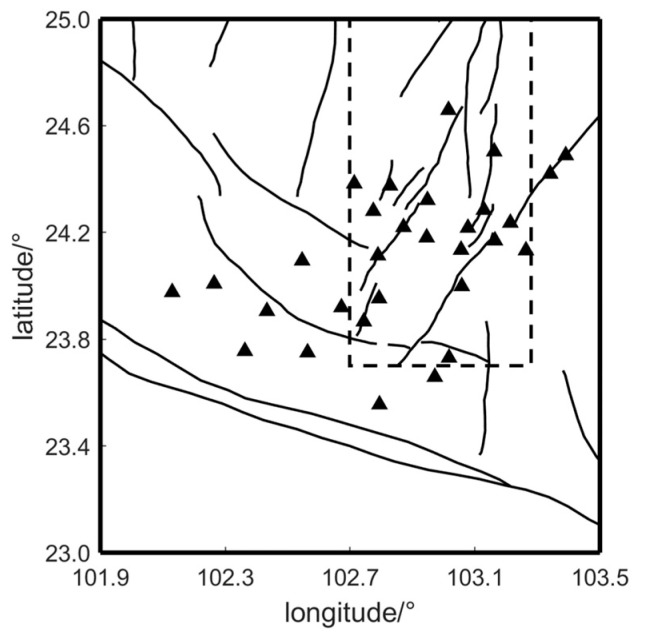
Distribution of selected measurement points (The solid lines within the dashed box indicate the southern segment of the Xiaojiang Fault Zone. Triangles represent measurement points.).

The measurement points in the observation area are equipped with buried fluxgate sensors from the Institute of Geophysics, China Earthquake Administration. These sensors record the H, D, and Z components of the geomagnetic field, with a sampling rate of 1 Hz, a measurement range of ±65,000 nT, and a resolution of 0.1 nT [19]. Due to varying levels of interference at different field measurement points, data accuracy is improved by selecting multiple points with high data quality and completeness for application. The 30 selected measurement points are approximately uniformly distributed on both sides of the major fault zones within the array area (see Figure 3), to better explore the subsurface electrical structure of the area between 23.4° N to 25° N latitude and 102° E to 103.5° E longitude.

The data selection period is from 1 February 2022, to 31 March 2022, Beijing time. Since the instruments were buried in the field during this period, external environmental influences on the data are unavoidable. Before data application, the raw second data must be preprocessed to remove external interference such as spikes and steps.

## 3. Regional 3D Subsurface Electrical Structure Inversion

Geophysical forward modeling assumes that the distribution of physical quantities such as conductivity and density inside the Earth is known, establishes different parameter models, and then obtains the distribution of various geophysical fields such as gravity field and electromagnetic field. In contrast, geophysical inversion processes and analyzes observation data from these fields to infer the distribution of physical parameters within the Earth, ultimately producing a representation of the subsurface material distribution.

For a typical forward modeling problem, this can be expressed in the following equation:

(1) Gm=d

where G is the forward operator, m is the model space, and d is the observed data. The inversion problem is then expressed as a process of solving the inverse operator $G^{-1}$,

(2) $$d=G^{-1}m=gm$$

Thus, geophysical inversion problems can be viewed as a process of solving mathematical and physical equations.

**Figure 4:**
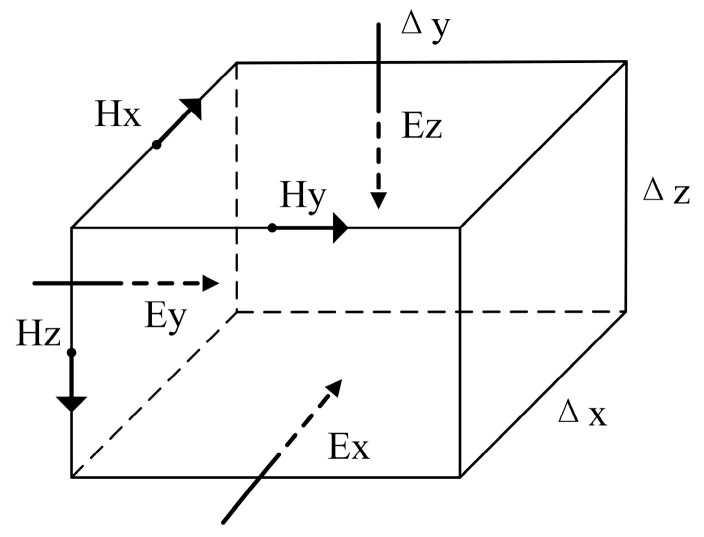
Schematic diagram of Yee staggered grid dissection.

In this paper, we use the ModEM 3D electromagnetic inversion program developed by Professor Egbert’s team [20] to perform subsurface electrical structure inversion calculations using the transfer functions obtained from 30 measurement points. Using the Yee [21] staggered grid finite difference method (shown in Figure 4), the study area is divided along the x, y, and z axes to obtain several grid cells. Each grid cell is assigned an (i,j,k) index and the resistivity of each grid cell is denoted as ρ(i,j,k).

For the electric and magnetic field strengths, the following vector equations are satisfied:

(3) $$\left\{\begin{array}{c} \nabla \times \frac{\mu_{0}}{\mu }\times \vec{E}+i\omega \mu_{0}\sigma \vec{E}=0\\ \nabla \times \rho \nabla \times \vec{H}+i\omega \vec{H}=0 \end{array}\right.$$

where σ is the electrical conductivity, which is the reciprocal of the resistivity ρ; $\mu_{0}$ is the vacuum permeability and μ is the magnetic permeability. By applying finite difference discretization to Equation (3), we obtain:

(4) $$\left\{\begin{array}{c} {[C}^{†}C+diag\left(i\omega \mu_{0}\sigma \left(m\right)\right)]e=0\\ {[C}^{†}diag\left(\rho \left(m\right)\right)C+diag\left(i\omega \mu \right)]e=0 \end{array}\right.$$

where $C^{†}$ and C represent the discrete approximation matrices for the vector convolution from faces to edges on the Yee grid and the vector convolution on the edges of the Yee grid, respectively; diag() denotes the diagonal elements of the parameter model matrix; and e represents the fundamental eigenvector of the initial space.

In the ModEM inversion program, the objective function to find the model parameter vector m is a regularized objective function based on gradient minimization. m represents the structural model of the subsurface conductivity. The expression of the objective function is:

(5) $$\Phi \left(m,d\right)={\left(d-f\left(m\right)\right)}^{T}C_{d}^{-1}\left(d-f\left(m\right)\right)+\nu {\left(m-m_{0}\right)}^{T}C_{m}^{-1}\left(m-m_{0}\right)$$

where $C_{d}$ denotes the covariance matrix of the observation error, $f\left(m\right)$ denotes the forward operator, $m_{0}$ denotes the initial parameter vector of the model, ν denotes the trade-off parameter, and $C_{m}$ denotes the covariance matrix of the model, with regularization defined through ${\nu^{-1}C}_{m}$.

By using the matrix similarity transformation $\tilde{m}=C_{m}^{-\frac{1}{2}}(m-m_{0})$ and $\tilde{f}\left(\tilde{m}\right)=f(C_{m}^{\frac{1}{2}}\tilde{m}+m_{0})$, we simplify the initial model vector $m_{0}$ and the covariance matrix $C_{m}$ in Equation (5). The following equation is obtained:

(6) $$\Phi \left(\tilde{m},d\right)={\left(d-\tilde{f}\left(\tilde{m}\right)\right)}^{T}\left(d-\tilde{f}\left(\tilde{m}\right)\right)+\nu {\tilde{m}}^{T}\tilde{m}={\left\|d-\tilde{f}\left(\tilde{m}\right)\right\|}^{2}+\nu {\left\|\tilde{m}\right\|}^{2}$$

By calculating $\tilde{m}$ using Equation (6), we then solve for m in Equation (5) as $m=C_{m}^{\frac{1}{2}}(\tilde{m}+m_{0})$ through the similarity transformation. During the ModEM solution process, the nonlinear conjugate gradient method (NLCG), which is computationally efficient and has fast iteration speeds, is employed [22,23,24,25].

**Figure 5:**
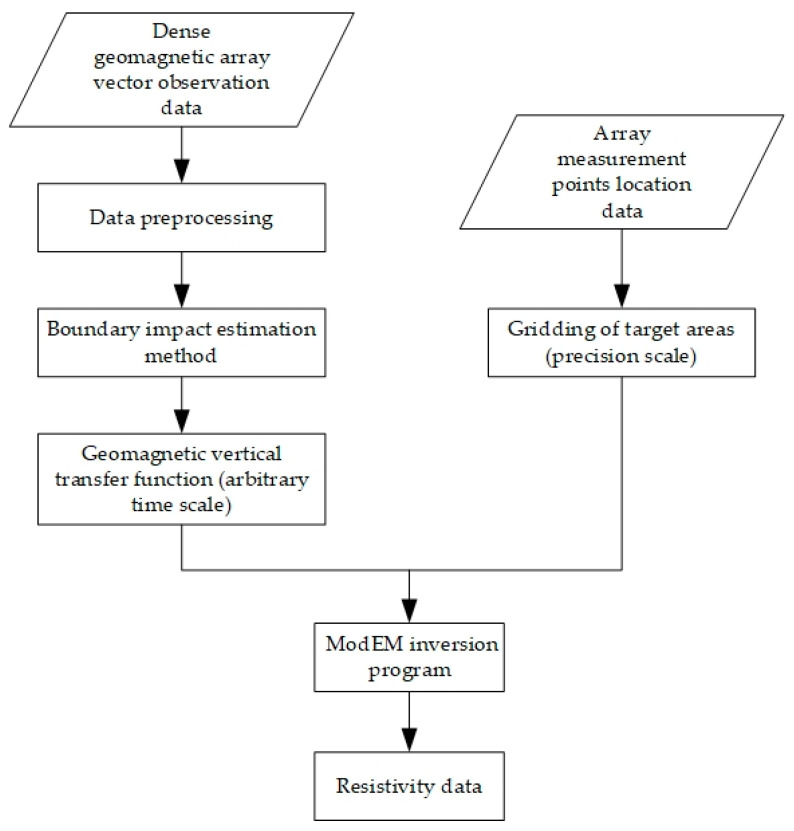
Dense geomagnetic array electrical structure data processing flow chart.

The overall data processing flow for the dense geomagnetic observation array is illustrated in the Figure 5. The process begins with preprocessing the observation data to remove interference and other noise. Then, on the basis of the traditional observation data processing methods, the dense geomagnetic array calculates the geomagnetic vertical transfer function by using the boundary impact estimation method at any desired time scale according to the requirements of the target model, and divide the regional grid model at a fine spatial scale. Finally, the ModEM method is employed to solve and calculate the subsurface electrical structure data.

## 4. Inversion Results of Subsurface Electrical Structure and Discussion

In the study area between latitudes 23.4° N to 25° N and longitudes 102° E to 103.5° E, assuming the terrain’s influence on the inversion results is negligible, this paper takes the point at latitude 23° N and longitude 101.9° E as the origin of a rectangular coordinate system. The latitudinal and longitudinal coordinates of each measurement point within the study area are converted to distance coordinates, and the area is divided into a grid. Within the central area covered by the dense geomagnetic observation points, the grid is uniformly spaced at 2.5 km intervals both north–south and east–west. Outside the central area, the grid intervals gradually increase.

**Figure 6:**
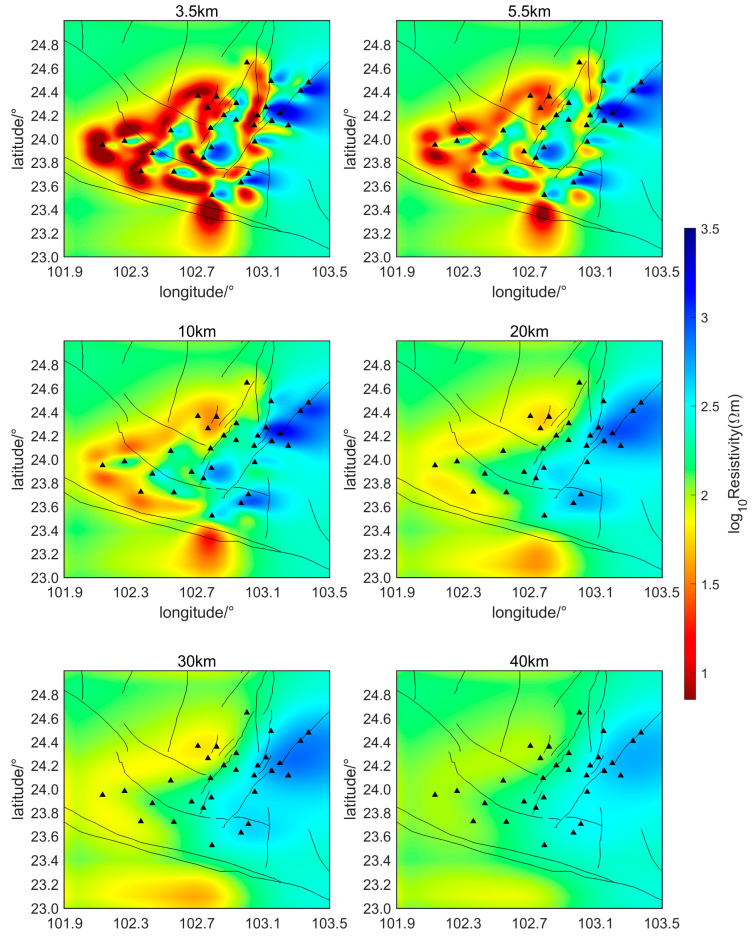
Relative subsurface resistivity of the study area from February 1 to 14 (Triangles represent measurement points).

**Figure 7:**
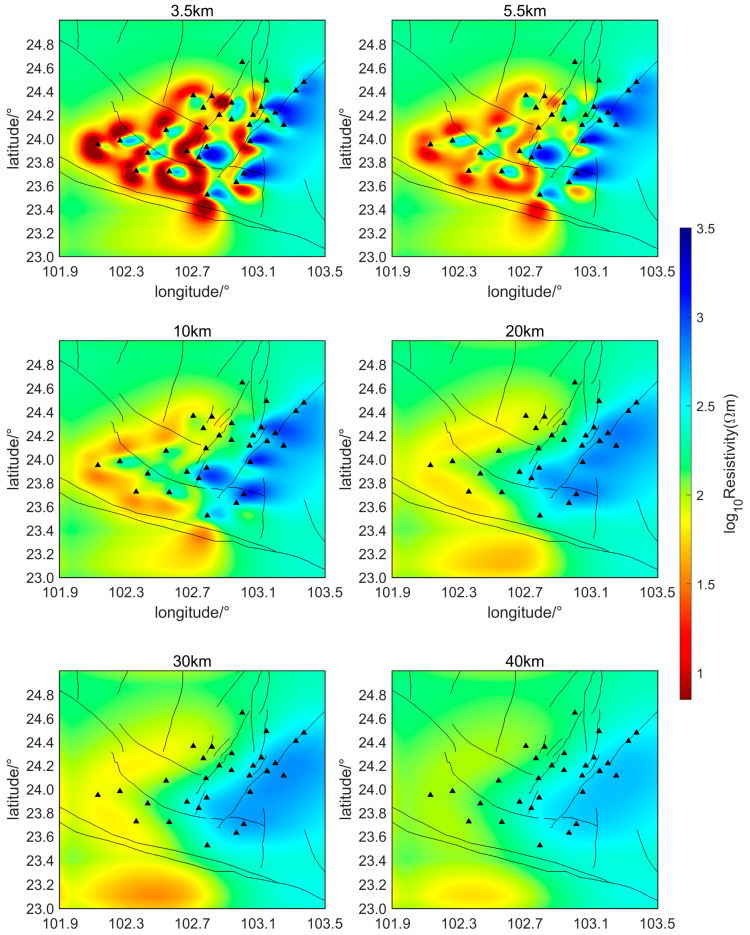
Relative subsurface resistivity of the study area from February 15 to 28 (Triangles represent measurement points).

**Figure 8:**
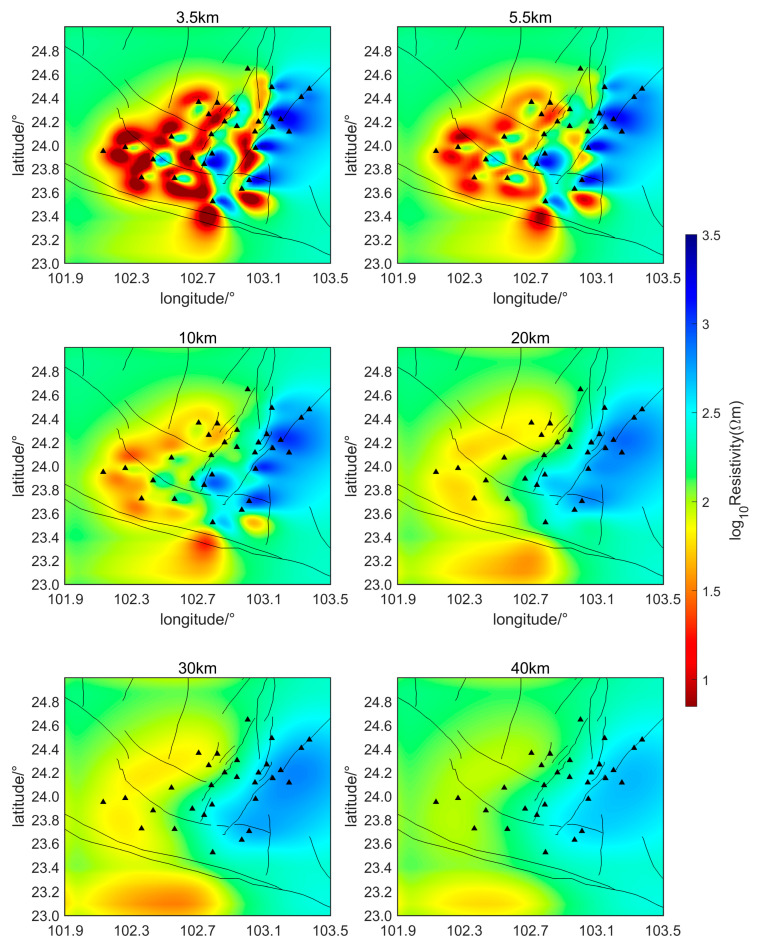
Relative subsurface resistivity of the study area from March 1 to 14 (Triangles represent measurement points).

**Figure 9:**
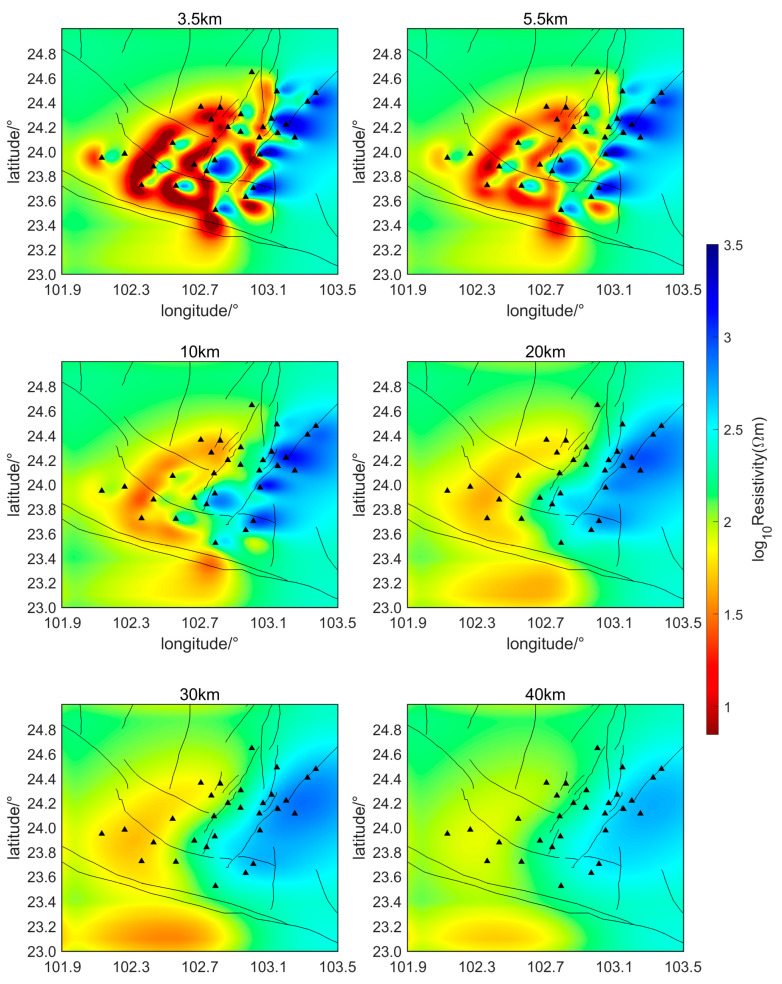
Relative subsurface resistivity of the study area from March 15 to 28 (Triangles represent measurement points).

The initial inversion model in this study employs a uniform half-space with a resistivity of 200 Ωm to perform the inversion calculations of the subsurface electrical structure over the two-month period, from 1 February 2022, to 31 March 2022. Horizontal slice maps of the relative subsurface resistivity inversion results at two-week intervals are shown in Figure 6, Figure 7, Figure 8 and Figure 9. The horizontal axis of the figures is longitude and the vertical axis is latitude. The black triangles indicate the locations of the measurement points, the black lines indicate the faults in the region. The slice depths in each picture are 3.5 km, 5.5 km, 10 km, 20 km, 30 km, and 40 km, respectively. Different colors represent different resistivities of the subsurface medium. Colors closer to red indicate lower resistivity, while colors closer to blue represent higher resistivity.

As can be seen from Figure 6, Figure 7, Figure 8 and Figure 9, during the seismic quiet period, the subsurface electrical structure of the study area remained basically stable, with no significant changes in magnitude. Spatially, the subsurface electrical structure is completely different on the two sides of the southern segment of the Xiaojiang Fault. Specifically, the resistivity on the west side of the fault is significantly lower than that on the east side, which is consistent with previous research [26]. Other scholars’ research has confirmed that, in areas where fault zones develop, there are often fractured zones containing water or other high-conductivity media. These fault zones can lead to anomalous changes in the stratigraphic structure, creating low-resistivity anomaly zones or electrical gradient zones [27]. The slice maps at various depths reveal that, within the study area, the subsurface electrical structure is characterized by large-scale low-resistivity bodies at depths ranging from approximately 3.5 to 30 km, with resistivities of around 10 Ωm.

Li Ran et al. set up a magnetotelluric survey line through this area. Their inversion results of the subsurface electrical structure also indicate that a large-scale, low-resistivity body exists beneath the Xiaojiang Fault Zone [28]. This body has a resistivity of approximately 10 Ωm and extends from a depth of about 3 to 20 km, running from west to east. The inversion results of this paper are consistent with their findings regarding the low-resistivity body. Some scholars believe that the existence of this electrical structure provides electrical evidence for the theory that the Sichuan–Yunnan rhombic block in the western part of the fault zone is being extruded southeastward during the northward wedging of the Indian Plate and the uplift of the Tibetan Plateau [29].

**Figure 10:**
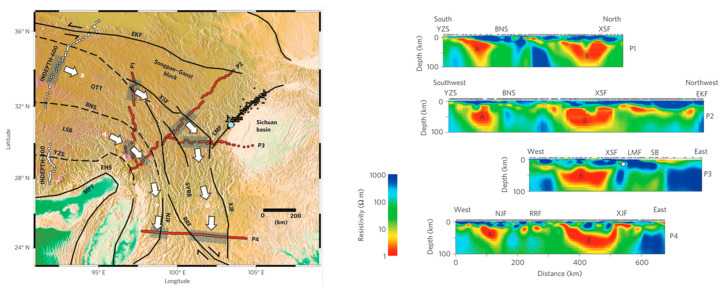
Magnetotelluric survey lines and electrical structure of the profile (cited from [30]) (A and B represent the two low-resistance body).

The magnetotelluric sounding study by Bai et al. [30] also showed (as shown in Figure 10) the existence of an east–west trending low-resistance body beneath the Xiaojiang Fault Zone. They believe that the existence of the low-resistance body in the active tectonic region is mainly due to the presence of water fluids and partial melting. This explanation is consistent with Yu Nian et al. (2022) regarding the low-resistivity anomalies in the Xiaojiang Fault Zone area [31].

Additionally, measurement of the geothermal heat flow indicates that the Xiaojiang Fault Zone has a relatively high geothermal heat flow value, averaging around 85 $mW/m^{2}$. The Moho temperature in the Xiaojiang Fault Zone region is approximately 965~1000 °C, while the lithosphere temperature is approximately 1528~1536 °C. The depth of the Curie surface is approximately 21 km within the region. The high temperatures suggest potential partial melting of rock layers, which could lead to a decrease in resistivity. Therefore, it is reasonable to infer that the low-resistivity anomalies observed in the Xiaojiang Fault Zone may be related to deep thermal processes [32,33]. 

The potential relationship between subsurface low-resistivity bodies and earthquake genesis is also a hot topic of research. Zhang Jihong et al. [34] proposed that there is usually a zone of abrupt conductivity changes near the earthquake preparation area, after analyzing historical major earthquakes in the Tan-Lu Fault Zone. After discussing the three-dimensional resistivity model of the area around the Ms6.5 Ludian earthquake, Cai et al. [24] found that aftershocks were mainly distributed in shallow high-conductivity regions surrounded by resistivity structures. They proposed that the presence of shallow high-conductivity zones might be a key factor in the severe damage and surface rupture caused by the Ludian earthquake. Tada-nori Goto et al. [35] studied the resistivity structure of the Atotsugawa Fault and found that the northern part of the Atotsugawa Fault is a high-resistivity zone, while the southern part is a low-resistivity zone. This is similar to the lateral resistivity differences observed in the southern segment of the Xiaojiang Fault Zone. They suggested that the lower crust appears as a conductive zone beneath the low-seismicity segment and is less conductive beneath the high-seismicity segment, and fluid is inferred to be the most likely cause of the conductive zone. The inversion results in this paper also show that there are obvious lateral variations in conductivity and large-scale, low-resistive body structures in the study area, which may be closely related to earthquake generation.

## 5. Conclusions

In order to obtain the geomagnetic field variation information with sufficient temporal and spatial resolution, monitor geomagnetic field changes in seismic hazardous areas such as active fault zones, capture seismomagnetic anomaly information in time, and provide data support and fundamental information for short-term earthquake prediction, this paper established a dense geomagnetic observation array in the southern segment of the Xiaojiang Fault Zone. A preliminary application research on the subsurface electrical structure of the study area is carried out by utilizing the high temporal and spatial resolution geomagnetic data from the dense array. During the 2-month observation period, no earthquakes with magnitudes above Ms 3.0 occurred in the area, indicating a seismic quiet period. Due to the long-term continuous observation of the array, the subsurface electrical structure model can be generated for any time length, significantly improving the temporal resolution compared to traditional mobile observation modes. Under the dense array observation mode, the spatial resolution of the inverted subsurface electrical structure is approximately 2.5 km, which represents a significant improvement in spatial resolution. The results indicate that during the seismic quiet period, the subsurface electrical structure of the region remains generally stable. The region features large-scale subsurface low-resistance bodies, and the electrical structures at both ends of the southern segment of the Xiaojiang Fault are markedly different. Future research could focus on monitoring geomagnetic data from measurement points near these low-resistance bodies to investigate possible changes in subsurface electrical structures associated with earthquakes.

## Data Availability

The data presented in this study are available on request from the corresponding author. The data are not publicly available due to privacy.
